# Photosynthetic alginate dressing enables sustained oxygen delivery through chloroplast-powered Hill reaction to promote angiogenesis and macrophage reprogramming for chronic wound healing

**DOI:** 10.1093/rb/rbag091

**Published:** 2026-05-21

**Authors:** Yifan Zhang, Lanqin Yu, Min Fang, Ying Zhou, Kun Zhong, Xinshuo Liu, Xiaoyun Liao, Lihua Li, Changren Zhou

**Affiliations:** Institute of Dermatology and Venereal Diseases, Affiliated Hospital of Guangdong Medical University, Zhanjiang 524001, China; College of Chemistry and Materials Science, Engineering Research Center of Artificial Organs and Materials, Jinan University, Guangzhou 510632, China; College of Chemistry and Materials Science, Engineering Research Center of Artificial Organs and Materials, Jinan University, Guangzhou 510632, China; College of Chemistry and Materials Science, Engineering Research Center of Artificial Organs and Materials, Jinan University, Guangzhou 510632, China; Institute of Dermatology and Venereal Diseases, Affiliated Hospital of Guangdong Medical University, Zhanjiang 524001, China; Institute of Dermatology and Venereal Diseases, Affiliated Hospital of Guangdong Medical University, Zhanjiang 524001, China; College of Chemistry and Materials Science, Engineering Research Center of Artificial Organs and Materials, Jinan University, Guangzhou 510632, China; Guangdong Provincial Key Laboratory of Spine and Spinal Cord Reconstruction, The Fifth Affiliated Hospital (Heyuan Shenhe People’s Hospital), Jinan University, Heyuan 517000, China; College of Chemistry and Materials Science, Engineering Research Center of Artificial Organs and Materials, Jinan University, Guangzhou 510632, China; College of Chemistry and Materials Science, Engineering Research Center of Artificial Organs and Materials, Jinan University, Guangzhou 510632, China

**Keywords:** sodium alginate, chloroplast, oxygen, Hill reaction, chronic wound healing

## Abstract

Chronic wounds are very difficult to heal due to persistent hypoxia, impaired angiogenesis and dysregulated inflammation. To address these problems, we developed a photosynthetic hydrogel dressing (SFCc) by integrating chloroplasts into an alginate-based matrix and optimizing the Hill reaction. Our system implements a dual photoprotection strategy using ferricyanide as an electron acceptor coupled with catalase (Cat), effectively neutralizing reactive oxygen species while sustaining the production of photosynthetic oxygen. The SFCc hydrogel demonstrated controlled release of oxygen and maintained >90% chloroplast viability for 72 h. In a pressure ulcer model, the hydrogel significantly decreased the expression of hypoxia-inducible factor-1α, enhanced angiogenesis with a 5.8-fold increase in the vascular density of CD31 and promoted the polarization of macrophages toward the regenerative M2 phenotype; these changes collectively accelerated wound healing. This chloroplast-powered platform represents a breakthrough in biomimetic oxygen delivery. Its unique function involves synchronizing oxygen supply with metabolic demand through light modulation while facilitating microenvironment-responsive release via pH-sensitive and Cat-sensitive mechanisms. Our photosynthetic approach overcomes the limitations of conventional oxygen carriers and offers a transformative strategy for chronic wound management and ischemic tissue repair.

## Graphical abstract

**Graphical Abstract:**
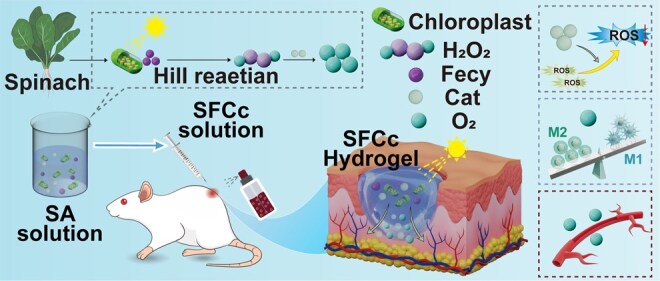

## Introduction

Chronic wounds are non-healing or delayed-healing wounds that persist beyond 1 month [1]. Their incidence has increased due to factors like aging populations and increased surgeries. Unlike normal wound healing (hemostasis, inflammation, proliferation and remodeling), chronic wounds (e.g. pressure ulcers, diabetic wounds) exhibit prolonged inflammation, leading to the consumption of large quantities of oxygen by immune cells and tissue necrosis, while impaired vasculature restricts oxygen supply, creating hypoxia [2]. This hypoxia increases the risk of infections, inhibits angiogenesis and epithelialization [3–5] and activates hypoxia-inducible factor-1α (HIF-1α), increasing the abundance of pro-inflammatory factors [6–8], further impairing wound healing. Oxygen therapy counteracts these effects by increasing oxygenation, angiogenesis and granulation tissue formation, particularly in pressure ulcers [9].

Hyperbaric oxygen (HBOT) and local approaches are the main techniques used in oxygen therapy for chronic wounds. Topical oxygen hydrogels and oxygen-releasing materials are promising alternatives to overcome the limitations of HBOT, which include the need for intermittent delivery and high cost [10, 11]. The main sources of oxygen are peroxides, such as hydrogen peroxide, calcium peroxide, copper peroxide and magnesium peroxide. They allow efficient release of oxygen and fast reaction rates [12–14]. However, peroxides have poor stability and decompose easily, their byproducts can interfere with the microenvironment, and the rapidly released oxygen may induce oxidative stress [15–19]. Photosynthetic oxygen production using microalgae or chloroplasts offers a biologically inspired solution [3, 20, 21]. Although the Hill reaction was discovered 87 years ago, its application in tissue repair remains limited primarily because maintaining photosynthetic activity in isolated chloroplasts is difficult. This necessitates the use of a biocompatible carrier that mimics the moist, soft environment of living organisms. Medical hydrogels, particularly sodium alginate (SA)-based hydrogels, serve as ideal scaffolds due to their water-retaining 3D network structure, ability to protect sensitive biomolecules through mild gelation, and optimal properties, including transparency for the transmission of light and permeability for the diffusion of oxygen [22–25]. These characteristics make them excellent candidates for supporting the viability of chloroplasts while meeting wound dressing requirements of maintaining a moist healing environment [26]. This integration of photosynthetic oxygen production with advanced hydrogel technology is highly effective in chronic wound treatment.

In this study, we developed an innovative *in situ* formable alginate hydrogel incorporating isolated chloroplasts (SFCc) capable of photolytic decomposition of water and generation of oxygen under illumination. By constructing a modified Hill reaction system using potassium ferricyanide (FeCy) as an electron acceptor and Cat to scavenge photosynthetic by-product peroxides, we significantly improved the viability of chloroplasts and enhanced the oxygen-producing capacity of the system. *In vitro* evaluations revealed the efficacy of the hydrogel in alleviating cellular hypoxia. Moreover, using a standardized mouse pressure ulcer model, we systematically investigated the therapeutic potential of this photosynthetic oxygen-generating system, demonstrating its wound healing capabilities through multiple evaluation metrics. The developed SFCc hydrogel represents a significant advancement in bioengineered wound dressings by combining the production of photosynthetic oxygen with the clinically favorable properties of hydrogels.

## Materials and methods

### Extraction and characterization of chloroplasts

Chloroplasts were extracted and characterized according to a previous publication [27], detailed descriptions have been provided in the supporting information.

### Construction of the Hill reaction oxygen production system

To construct the Hill reaction oxygen production system, tris-HCl (50 mmol/L), MgCl_2_ (50 mmol/L), NaCl (100 mmol/L) and chloroplasts (1.5, 3, 6, 12 and 24 μg/mL) were dissolved in deionized water to prepare the Hill reaction solution. Fe ion compounds, including FeCl_3_ (1 mmol/L) and FeCy (1 mmol/L), were weighed and added as oxidants to the Hill reaction solution for subsequent characterization [28].

Different light intensities (100 W, 500 W and 800 W) were set with a chloroplast concentration of 24 μg/mL and the dissolved oxygen (DO) content was monitored over 30 min to screen for a suitable light intensity. To determine the optimal iron-containing compounds, their concentrations, and the appropriate chloroplast content, Hill reaction solutions containing chloroplasts at varying concentrations (3, 6, 12 and 24 μg/mL) were supplemented with FeCl_3_ and FeCy (1 mmol/L). The solutions were then irradiated under a 500-W iodine-tungsten lamp in a nitrogen environment for 20 min [29, 30]. The DO levels were measured using a dissolved oxygen meter (JPB-607A, INESA, China). Additionally, the chloroplasts oxygen-producing systems were subjected to sonication in an ultrasonic instrument (240 W, 20 min) to evaluate the impact of ultrasound on oxygen production. Following sonication, the oxygen production of the Hill reaction was measured again using the same procedure.

### Preparation and characterization of hydrogels

A precursor solution containing 50 mmol/L Tris-HCl, 50 mmol/L MgCl_2_ and 100 mmol/L NaCl was added to a 2% (w/v) SA solution. After precursor solution, 1 mmol/L FeCy was introduced. Chloroplasts (12 μg/mL) and Cat (0.5%) were then added successively until the FeCy was completely dissolved. The mixture was subjected to ultrasonication for 20 min at 240 W to prepare the precursor solution. The precursor was then poured into a mold, and a 4% (w/v) CaCl_2_ solution was sprayed onto it to induce gelation, resulting in the formation of alginate hydrogel, which contained FeCy, Cat and chloroplasts (SFCc), while recording the hydrogel formation process.

Using identical preparation protocols, three hydrogels were synthesized: SFC containing FeCy and Cat; SFc containing FeCy and chloroplasts; and SF containing solely FeCy. In addition, 4% (w/v) CaCl_2_ solution was sprayed directly on 2% (w/v) SA solution to form SA hydrogel.

The prepared SFCc hydrogel was adhered to the surfaces of steel, plastic, skin and wood, and photographs were taken to document its adhesive performance on these different materials. The rheological properties of hydrogels were tested using a rotational rheometer (Kinexus Pro, UK) at 25°C. The precursor was injected onto the center of the rheometer stage, followed by spraying with CaCl_2_ to induce gelation. The upper clamp was quickly lowered to contact the hydrogel surface and started scanning. The tests were performed at a fixed strain of 1% while varying the frequency from 0.1 to 100 Hz.

The hydrogels were enclosed in a dialysis bag and immersed in a 15-mL centrifuge tube with 10 mL of deionized water. The tube was then placed in a constant temperature water bath at 37°C for 30 min. During this period, the pH of the solution was measured and recorded in real-time using a pH meter (PHS-3E, LEICI).

The SFc and SFCc hydrogels were enclosed in dialysis tubing in a 15-mL centrifuge tube and exposed to 10 mL PBS (pH = 7.4). After 30 min of illumination in a nitrogen atmosphere, the DO concentration in the sample medium at each time point was measured using a dissolved oxygen meter.

The SFc, SFC and SFCc hydrogels were sealed in 15 mL centrifuge tubes containing 10 mL of PBS solution with 100 μmol/L H_2_O_2_. Under a nitrogen atmosphere, they were exposed to light for 2 h, followed by incubation in the dark for 10 h. This 12-h cycle was repeated, and the cumulative oxygen release was measured over a period of 48 h.

### Cell culture experiments

The section on cell experiments is included in the supporting information.

### Assessment of pressure ulcer wound healing

All animal experiments complied with the National Research Council’s Guide for the Care and Use of Laboratory Animals and were reviewed and approved by the Animal Ethics Committee of Ruige Biotechnology (Approval No. IACUC 20240109-001), China.

An ischemic pressure ulcer model was established by applying a magnetic clamp (diameter: 10 mm) to compress the mouse skin, inducing localized ischemia. The compression was maintained for 4 h, after which the magnet was removed to permit reperfusion. This process was continued for 3 d to form an ischemic ulcer. The ulcers were photographed at specific time points and then covered with the appropriate treatment material. The wound edges were secured using a 3M^TM^ medical vent patch. After recovery from anesthesia, the mice were individually placed in separate cages.

Following successful ulcer modeling, mice were randomly divided into three groups (*n* = 6 per group): Control group (treated with alginic acid hydrogel), SFc hydrogel group (treated with SFc hydrogel) and SFCc hydrogel group (treated with SFCc hydrogel). All hydrogels were standardized to dimensions of Ø = 12 mm × 2 mm. The hydrogels were changed every 48 h throughout the treatment period. Weigh the mice every 2 d after surgery and record the weight changes.

#### Measurement of wound healing

During the wound treatment process, the 3M medical breathable film was removed at specific time points (0, 3, 7 and 14 d); the wound diameter was measured; and photographs of the wound area were captured with a camera. Wound area was then calculated using ImageJ software. Healing efficacy was calculated through the wound healing rate (η), according to the following equation:


$$\mathrm{Wound}\;\mathrm{healing}\;\mathrm{rate}\;(\%)=\frac{S_{0}-S_{t}}{S_{0}}\times 100\%.$$

Here, S_0_ and S_t_ represent wound areas at 0 d and specific time points, respectively.

#### Histopathological, immunohistochemical and immunofluorescence analyses

After euthanasia, wound tissues were collected on 3, 7, 10 and 14 d, washed with PBS and fixed in 4% paraformaldehyde solution for subsequent histopathological, immunohistochemical and immunofluorescence analyses.

Paraffin-embedded specimens were processed with hematoxylin & eosin (H&E) and Masson’s trichrome staining kits (Servicebio) following standard protocols. Immunofluorescence staining targeted vascularization (CD31), myofibroblast differentiation (α-SMA) and hypoxia response (HIF-1α), while immunohistochemical analysis characterized macrophage polarization markers (CD163, CD80, IL-10 and IL-1β). All stained sections were imaged using a Leica Dmi1 inverted microscope under standardized optical configurations. The organs (heart, liver, spleen, lung and kidney) were collected for H&E staining to observe whether the material caused toxic reactions in mice.

### Statistical analysis

All data were analyzed using Graphpad Prism 7.0 software, with one-way ANOVA applied to determine significant differences among groups. The results are expressed as the mean ± standard deviation (SD). Significant differences were marked as follows: *P < 0.05, **P < 0.01 and ***P < 0.001.

## Results

### Characterization of chloroplasts

**Figure 1:**
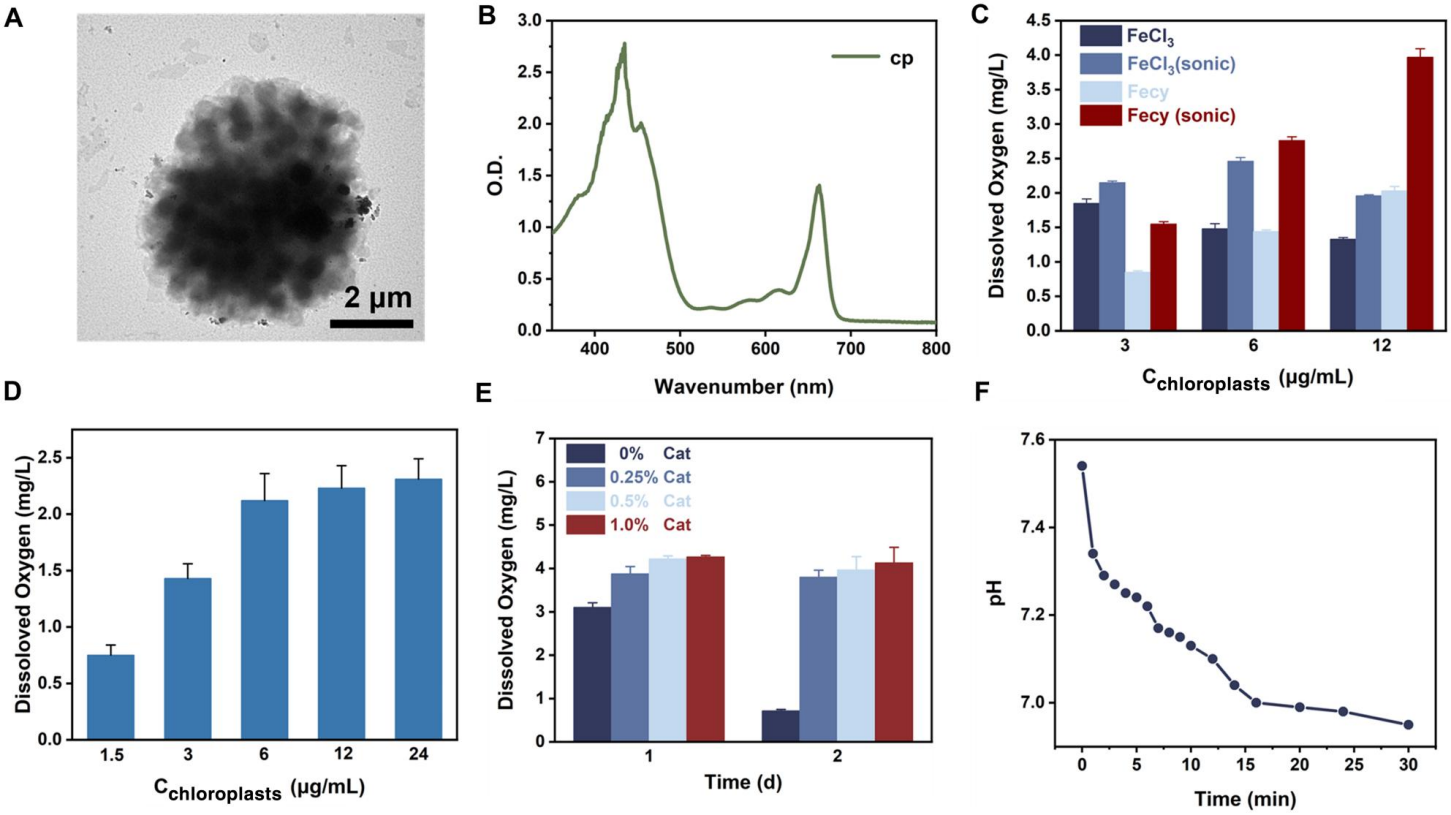
Characterization of chloroplasts and oxygen production. (**A**) TEM image of a chloroplast. (**B**) The full-band UV-Vis absorption spectrum of chloroplasts is shown. (**C**) Oxygen production of FeCl_3_ and FeCy in reactions with chloroplasts at different concentration gradients is illustrated. (**D**) The relationship between oxygen production and chloroplast concentration is shown. (**E**) Oxygen production of FeCy and chloroplasts under different concentrations of Cat is illustrated. (**F**) The changes in pH during the Hill reaction are shown (*n* = 3).

The optical microscopy images revealed that the extracted chloroplasts were evenly distributed and had a soft oval shape (Supplementary Figure S1). The Transmission Electron Microscope (TEM) images confirmed that the extracted chloroplasts were 2–4 μm wide and 4–10 μm long; they were elliptical like chloroplasts, as reported in previous studies (Figure 1A) [31]. Additionally, a full-band scan of the chloroplast extract using a UV-Vis spectrophotometer revealed a distinct absorption peak at 650–680 nm (Figure 1B). This peak was attributed to the high chlorophyll content in chloroplasts, which allows them to absorb most of the red light while reflecting green light. The scan also revealed strong absorption in the ultraviolet wavelength range, confirming the presence of chlorophyll and its light-absorbing properties. Chloroplasts drive the release of oxygen using light. Experiments demonstrating the effects of different light intensities on oxygen production behavior revealed that as light intensity increases, DO concentration increases and the rate of oxygen release also increases. However, when the light intensity was increased to 800 W, DO rapidly decreased after reaching its peak. This occurred because high light intensity induces photothermal effects or photooxidative stress, damaging the structure of chloroplasts and inactivating photosynthetic functions. Therefore, 500 W was selected as the light intensity for subsequent studies (Supplementary Figure S2).

### Construction and optimization of the Hill reaction system

To analyze the oxygen production capacity and determine the optimal conditions of the Hill reaction system in chloroplasts, equimolar concentrations of FeCl_3_ and FeCy were added to the Hill reaction solution. The DO results revealed that when the chloroplast concentration was 3 μg/mL, FeCl_3_-based systems produced higher concentrations of DO initially (1.8 mg/L) than the FeCy counterparts (0.9 mg/L), with sonication increasing outputs to 2.1 mg/L and 1.6 mg/L, respectively (Figure 1C). As the concentration of chloroplasts increased to 6 μg/mL, the FeCl_3_ systems produced lower concentrations of DO (1.4 mg/L), while the sonicated FeCy system produced 2.7 mg/L DO, outperforming all treatments at this concentration. The implementation of FeCy showed marked concentration sensitivity. Optimal performance emerged at 12 μg/mL, where the combination of sonication and FeCy generated the highest DO levels (4.0 mg/L), representing a 90.5% improvement over non-sonicated FeCy controls.

To determine the optimal chloroplast concentration, DO values were measured over 20 min (Figure 1D). We found a significant positive correlation between oxygen production and chloroplast concentration up to 12 μg/mL. Beyond this critical threshold, oxygen output plateaued, although chloroplast concentrations increased. Therefore, 12 μg/mL was established as the operational concentration of chloroplast for subsequent experiments. Additionally, Cat concentration was found to be a critical determinant of oxidative stability. Initial DO levels exhibited concentration-dependent stabilization, ranging from 3.0 mg/L (0% Cat) to 4.2 mg/L (1.0% Cat) on the first day (Figure 1E). On the second day, uncatalyzed systems (0% Cat) showed catastrophic oxygen depletion (ΔDO=−83.3%), plummeting to 0.5 mg/L, whereas Cat-supplemented systems maintained oxygenation near peak concentrations (3.8–4.2 mg/L, ΔDO≤−9.5%). We found that 1.0% of Cat achieved complete DO retention (4.2 mg/L sustained), outperforming lower concentrations of Cat. However, even minimal Cat supplementation (0.25%) preserved 92.7% of initial DO, which was higher compared to the 16.7% retention of DO in controls; this indicated that Cat has threshold-independent protective effects. Chloroplasts undergo the Hill reaction in the presence of light, photolyzing water to produce oxygen. In this process, the O^2−^ in the H_2_O molecule loses electrons and is converted into ground-state oxygen, while the H^+^ ions remain unchanged. Therefore, in this reaction, every two H_2_O molecules produce one molecule of O_2_ and four H^+^ ions, leading to a decrease in the pH of the system due to the accumulation of H^+^ ions. Very low pH can negatively affect the photosynthetic activity of chloroplasts and may also hinder the Hill reaction oxygen-producing system in organisms, medical settings and other fields. The pH of the Hill reaction decreased rapidly at the initial stage, but this decrease gradually slowed down as the duration of exposure to light increased, and after reducing to 7.0, there were no further changes. This pH is within the range suitable for most biological systems and does not denature the photosynthetic proteins in chloroplasts (Figure 1F). High concentrations of Fe ions induce mitochondria to produce ROS and trigger ferroptosis, a form of iron-dependent regulated necrosis, in cells [32]. The results of the CCK-8 assays revealed concentration-dependent biocompatibility of FeCy (Supplementary Figure S3A and B). At high concentrations (10 mmol/L), FeCy exhibited significant cytotoxicity. However, at low concentrations (0.1 and 1 mmol/L), FeCy demonstrated high biocompatibility coupled with proliferative effects on endothelial cells, which serve as critical drivers of neoangiogenesis. This dual functionality aligns with the established role of Fe in the activation of endothelial cells via HIF-1α/VEGF signaling potentiation. Although 10 mmol/L FeCy had a greater oxygen generation capacity, the 1 mmol/L formulation emerged as the therapeutic optimum. These findings delineate a concentration window (0.5–2 mmol/L) where FeCy synergistically balances catalytic efficiency with biosafety; therefore, FeCy can be integrated into oxygen-delivery systems for regenerative medicine applications.

### Characterization of SFCc hydrogels

**Figure 2:**
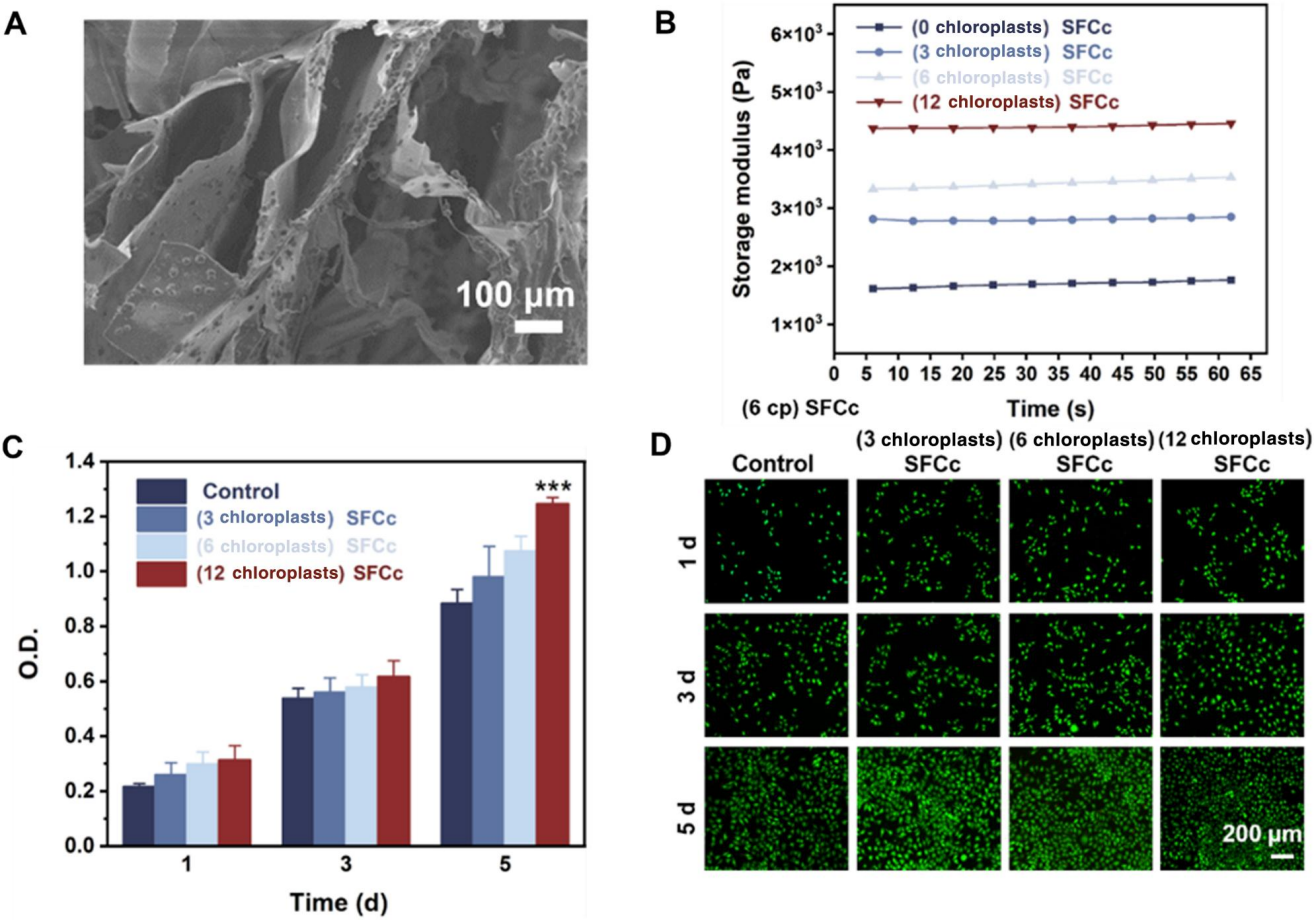
Characterization and biocompatibility of SFCc hydrogels. (**A**) The SEM image shows an SFCc hydrogel. (**B**) Rheological properties of SFCc hydrogels containing different concentrations of chloroplasts (0, 3, 6 and 12 μg/mL) are illustrated. (**C**) Cellular viability of L929 cells treated with SFCc hydrogels is presented. (**D**) Cytotoxicity test of SFCc hydrogels containing different concentrations of chloroplasts was conducted (***P < 0.001; *n* = 3).

The process used to prepare the SFCc hydrogels was convenient and rapid. The precursor solution was applied to the wound site and formed a gel after it was cross-linked via calcium ion spraying (Supplementary Video S1, S2). The results of the adhesion test showed that SFCc exhibited good adhesive properties on surfaces such as steel, plastic, skin and wood (Supplementary Figure S4). The Scanning Electron Microscope (SEM) images revealed that SFCc hydrogels had an interconnected microporous network, which facilitates efficient exudate absorption while maintaining gas diffusion pathways essential for aerobic wound healing (Figure 2A). The surfaces of SA, SFc and SFCc materials were porous. The incorporation of FeCy led to a uniform distribution of iron throughout the gel. Magnesium, an element found in chloroplasts, was distributed within both SFc and SFCc materials. These results indicate that chloroplasts and FeCy were successfully introduced into the hydrogel (Supplementary Figure S5). Rheological profiling revealed concentration-dependent mechanical reinforcement (Figure 2B). While base alginate hydrogels crosslinked with Ca^2+^ had a storage modulus (G') of about 1.5 ± 0.2 kPa, chloroplast incorporation induced progressive stiffening, increasing the G′ to 4.2 ± 0.3 kPa at 12 μg/mL. This mechanical enhancement mainly occurred due to chloroplast-derived hydrogen bonding and the alginate matrix, forming a dual-crosslinking mechanism (ionic Ca^2+^-guluronate bridges and supramolecular interactions). The optimized viscoelasticity of the hydrogel (G′ ≈ 4.2 kPa) aligns with the biomechanical requirements of the human dermis (2–10 kPa), ensuring that it is compatible with dynamic tissue environments. Moreover, time-dependent rheological tests confirmed structural stability, with G′ retaining 94% ± 3% of its initial value over 60 s.

At the wound site, hypoxia causes oxidative stress, negatively affecting the activity of chloroplasts. Therefore, the effect of H_2_O_2_ on chloroplast activity within hydrogels needs to be investigated. For this, chloroplasts and SFCc hydrogels were exposed to H_2_O_2_ solutions to test their ability to produce oxygen. H_2_O_2_ substantially decreases the oxygen production capacity of chloroplasts, while the SFCc hydrogel maintains a higher level of activity. This difference can be attributed to the presence of Cat in the SFCc hydrogel, which neutralizes ROS, thereby mitigating the detrimental effects of H_2_O_2_ on the functions of chloroplasts. In the absence of H_2_O_2_, the oxygen-releasing capacity of the SFCc hydrogel decreases (Supplementary Figure S6A). The results of the oxygen release performance test for the SFCc hydrogels indicated that the SFc hydrogels exhibited a significantly lower oxygen release capacity after 15 min of illumination compared to the SFCc hydrogels. The buffering capacity of the SFCc hydrogel ensures the presence of a stable environment that supports sustained function of chloroplasts, whereas the SFc hydrogel lacks this protective mechanism, leading to a reduction in oxygen production (Supplementary Figure S6B). The oxygen release results indicated that with 2-h light exposure within a 12-h period, the photosynthetic activity of chloroplasts significantly increased the DO content in the SFc and SFCc groups. Due to the action of Cat, the DO level in the SFC group also showed a notable improvement. Under the combined effects of chloroplasts and Cat, the SFCc group demonstrated the most pronounced enhancement in DO content (Supplementary Figure S7).

The results of the CCK-8 assays demonstrated sustained cytocompatibility and proliferation-promoting effects of SFCc hydrogels across a chloroplast concentration gradient (Figure 2C). At all timepoints, the OD values of the SFCc treatment groups were higher than those of the control group, with increases of 15–25% (Day 1), 25–35% (Day 3) and 35–60% (Day 5), respectively. Fluorescence microscopy images confirmed this bioactivity gradient, showing that progressive increases in cell density were correlated with both chloroplast concentration and culture duration (Figure 2D). On Day 5, the 12 μg/mL chloroplasts group showed the most significant cell growth, which indicated that the material could enhance cellular activity.

### Regulation of hypoxic state in cells by SFCc hydrogels

**Figure 3:**
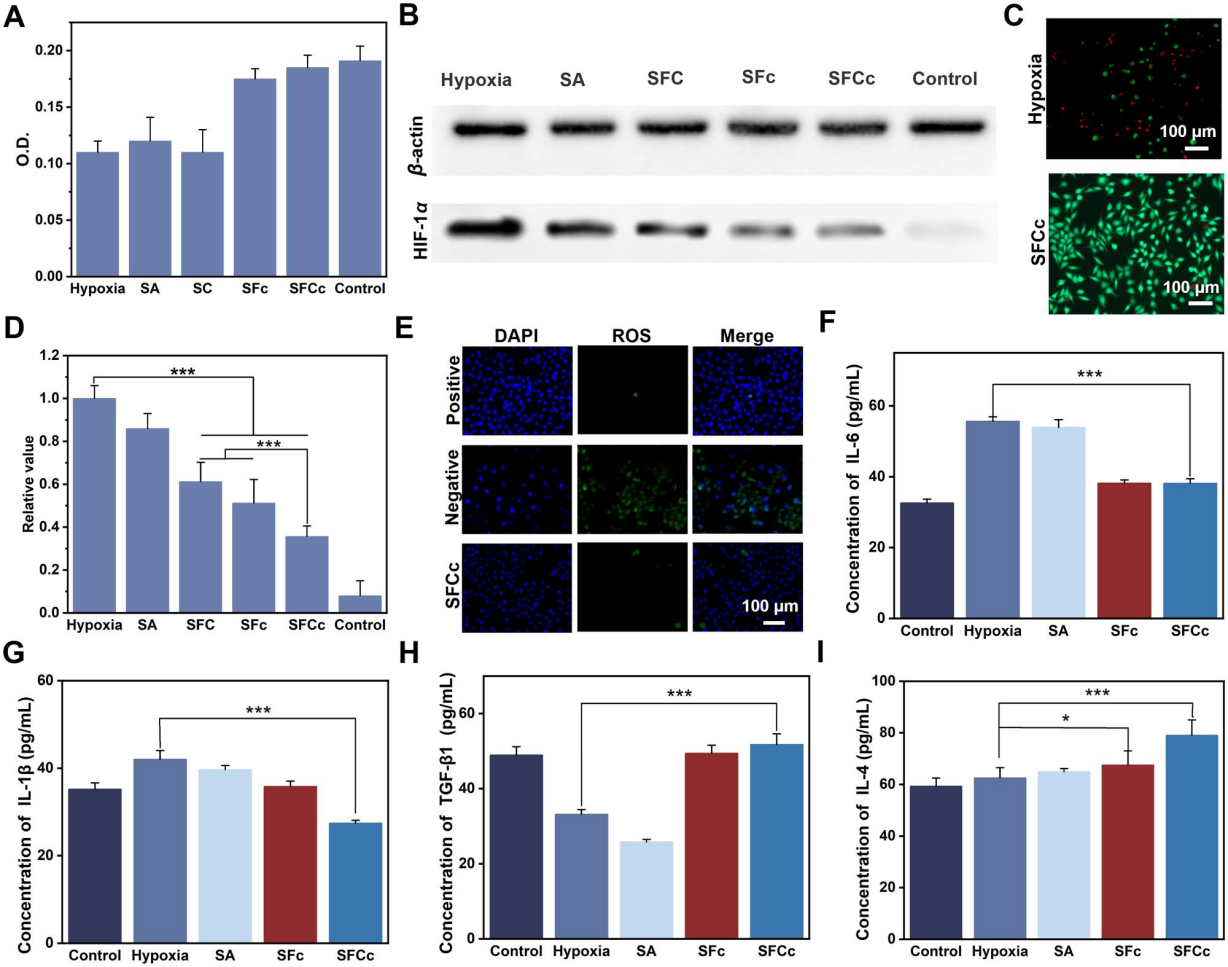
The effects of hydrogel treatment on L929 and RAW264.7 cells are presented. (**A**) Protein content, (**B**) WB bands, (**C**) cell viability staining, (**D**) quantitative statistics of WB grayscale values and (**E**) fluorescence staining of intracellular ROS scavenging of L929 cells after treatment with hydrogels are illustrated. ELISA was performed to determine the levels of (**F**) IL-6, (**G**) IL-1β, (**H**) TGF-β1 and (**I**) IL-4 in RAW264.7 cells after hydrogel treatment (*P < 0.05, ***P < 0.001; *n* = 3).

To mimic the hypoxic injury environment, L-929 cells were exposed to CoCl_2_-induced hypoxic stress, a well-established model for HIF-1α stabilization [33]. The results of the quantitative analysis revealed significant depletion of intracellular proteins in hypoxic controls (42% reduction compared to normoxia) and SA hydrogel groups (38% reduction). In contrast, SFc and SFCc hydrogels maintained protein levels close to the baseline (± 5%), which indicated that they could mitigate hypoxic stress through photosynthetic oxygen generation (Figure 3A). The performance of the SFc and SFCc groups was comparable to that of the normoxia control, which highlighted that these hydrogels could serve as effective oxygen-supplying biomaterials. The results of the Western blotting analysis showed that hypoxia-induced conditions triggered robust stabilization of HIF-1α, consistent with oxygen-dependent proteasomal degradation mechanisms (Figure 3B and D). SFC, SFc and SFCc hydrogels significantly decreased HIF-1α levels. Quantitative analysis revealed that SFCc had the strongest inhibitory effect on the expression of HIF-1α, and the strength of its inhibitory effect was significantly different from that of SFC and SFc. The morphological features and proliferation levels of cells were also assessed to evaluate relative cell viability. The cell activity in the hypoxia group was very low, and the green fluorescence intensity was weak, indicating poor cell viability (Figure 3C). In contrast, treatment with SFCc hydrogels significantly increased the green fluorescence intensity, indicating desirable cell morphology, distinct cell antennae and a significant increase in cell density.

### Antioxidant properties of SFCc hydrogels

We systematically evaluated the ROS-clearance capacity of SFCc hydrogels by conducting scavenging assays targeting four major oxidative radicals (Supplementary Figure S8). The antioxidant efficacy was dose-dependent, and chloroplast concentration (3–12 μg/mL) was positively correlated with radical elimination rates. Cat supplementation induced radical-specific modulation: hydroxyl (OH) and superoxide (O^2−^) scavenging decreased slightly, while H_2_O_2_ and DPPH neutralization increased significantly. This differential effect can be attributed to the dual mechanistic roles of Cat: direct transfer of electrons to stabilize DPPH radicals via the inhibition of the Fenton reaction, and heme prosthetic group-mediated catalytic decomposition of H_2_O_2_ (2H_2_O_2_ → 2H_2_O + O_2_). These results indicated that Cat could serve as a redox modulator in SFCc systems, selectively increasing peroxide scavenging while maintaining baseline free radical homeostasis, a critical feature for preventing oxidative damage cascades in chronic wounds.

We quantified intracellular oxidative stress modulation using DCFH-DA fluorescence in H_2_O_2_-challenged L929 fibroblasts (Figure 3E). The negative control group, stimulated with H_2_O_2_, exhibited a high level of green fluorescence, indicating the presence of high levels of ROS. Treatment with SFCc hydrogels reduced H_2_O_2_-induced fluorescence, and the treated cells showed near-baseline ROS levels comparable to the ROS levels found in unstressed cells. These findings confirmed that SFCc hydrogels can serve as potent intracellular redox regulators, capable of maintaining physiological ROS homeostasis under pro-oxidative stress conditions.

### Modulation of the polarization of macrophages by SFCc hydrogels under hypoxic conditions

Chronic wound healing is critically impeded by hypoxia-driven M1 macrophage polarization, a pro-inflammatory phenotype that predominates in oxygen-deprived environments. Flow cytometric analysis revealed that under hypoxic conditions, 75% of macrophages adopted the M1 phenotype (Q4 quadrant), while SFCc hydrogel treatment significantly reduced M1 polarization to 58% (Supplementary Figure S9). Quantitative cytokine analysis demonstrated SFCc hydrogel’s capacity to reprogram inflammatory signaling cascades in hypoxic microenvironments via ELISA (Figure 3F–I). The levels of pro-inflammatory mediators IL-6 and IL-1β decreased by 42% and 33% compared with hypoxic controls. Conversely, anti-inflammatory factors TGF-β1 and IL-4 were markedly enhanced by 100% and 55%, indicating robust M2 macrophage polarization.

### Cellular migration and pro-angiogenic signaling mediated by SFCc hydrogels

**Figure 4:**
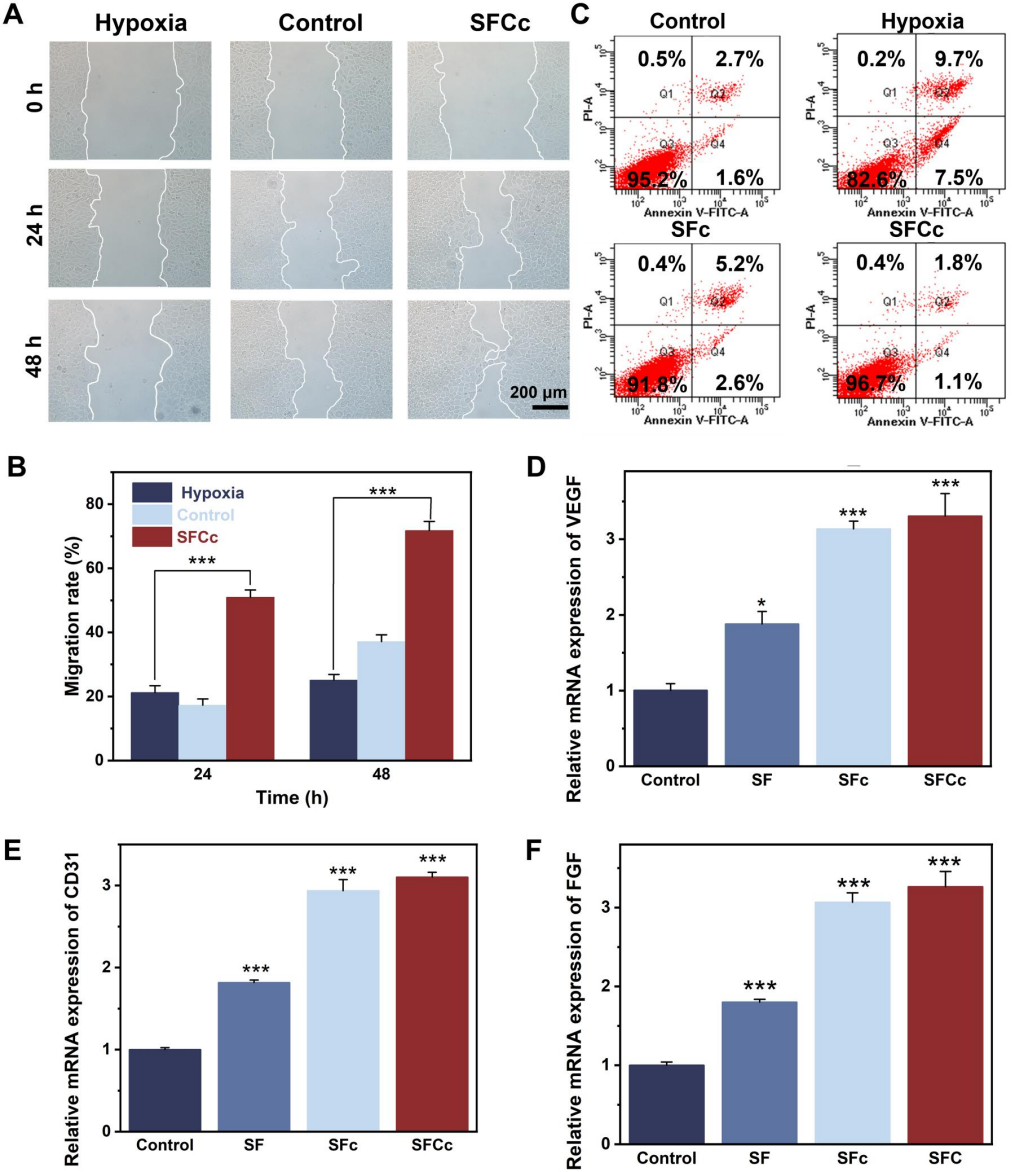
Effects of SFCc hydrogels on vascularization. (**A**) Migration images and (**B**) migration rate of HUVECs after treatment with hydrogels. (**C**) Apoptosis of HUVECs after treatment with hydrogels is shown. The expression of (**D**) VEGF, (**E**) CD31 and (**F**) FGF genes are shown (*P < 0.05, *** *P* < 0.001; *n* = 3).

Lack of vascularization makes it difficult to heal chronic wounds. Angiogenesis is closely related to the proliferation and migration of endothelial cells. Our results revealed that SFCc hydrogels have a greater capacity to induce migration (Figure 4A and B). At 24 h post-treatment, SFCc hydrogels accelerated the migration of HUVECs by 2.5-fold versus hypoxia controls, surpassing normoxic baselines (15%) through oxygen-dependent metabolic reactivation. By 48 h, SFCc achieved 70% migration, which was double the migration rate recorded under normoxic conditions. This increase occurred due to dual mechanisms: sustained release of oxygen (4.2 mg/L), which restored ATP-dependent cytoskeletal remodeling, and Fe³^+^ in the SFCc hydrogels, which mediated the activation of endothelial cells.

The results of the q-PCR analysis of angiogenic markers (Figure 4D–F) demonstrated that they were upregulated across hydrogel formulations. SF hydrogels increased the levels of VEGF (1.8-fold), CD31 (1.7-fold) and FGF (1.6-fold) compared to the controls. SFc hydrogels further upregulated their expression, while SFCc hydrogels achieved peak induction (VEGF: 3.3-fold, CD31: 3.1-fold and FGF: 3.3-fold). This oxygen-gradient-dependent amplification implicates the potentiation of the HIF-1α/VEGF pathway, synergized by the role of Fe^3+^ in the activation of endothelial nitric oxide synthase. Thus, the combinatorial oxygen-iron delivery system establishes a pro-angiogenic niche, thereby promoting neovascularization.

### Delaying cellular apoptosis in hypoxic conditions by SFCc hydrogels

The flow cytometry was performed to evaluate apoptosis (Figure 4C). The results showed that the number of cells in Q2 (late apoptosis) and Q4 regions (early apoptosis) of the hypoxia group was significantly higher than that in the control group. In the SFc group, the number of cells in the Q4 region was significantly lower, which suggested that early apoptosis was partly inhibited. In the SFCc group, the number of cells in the Q2 and Q4 regions decreased significantly. These findings indicate that SFCc hydrogels have a delaying effect on both the early and late stages of apoptosis. Oxygen strongly contributes to maintaining normal physiological activities in cells, and variations in its concentration influence apoptosis. Appropriate oxygen levels help stabilize mitochondrial function, balance energy metabolism and prolong cell survival. Disruption of oxygen homeostasis, whether in the form of sustained hypoxia or hyperoxia exposure, may promote apoptosis through pathways such as the induction of abnormal accumulation of ROS, disruption of mitochondrial membrane potential, modulation of the expression of proteins in the Bcl‑2 family and activation of the caspase cascade.

### Enhanced wound healing and tissue regeneration by SFCc hydrogels

**Figure 5:**
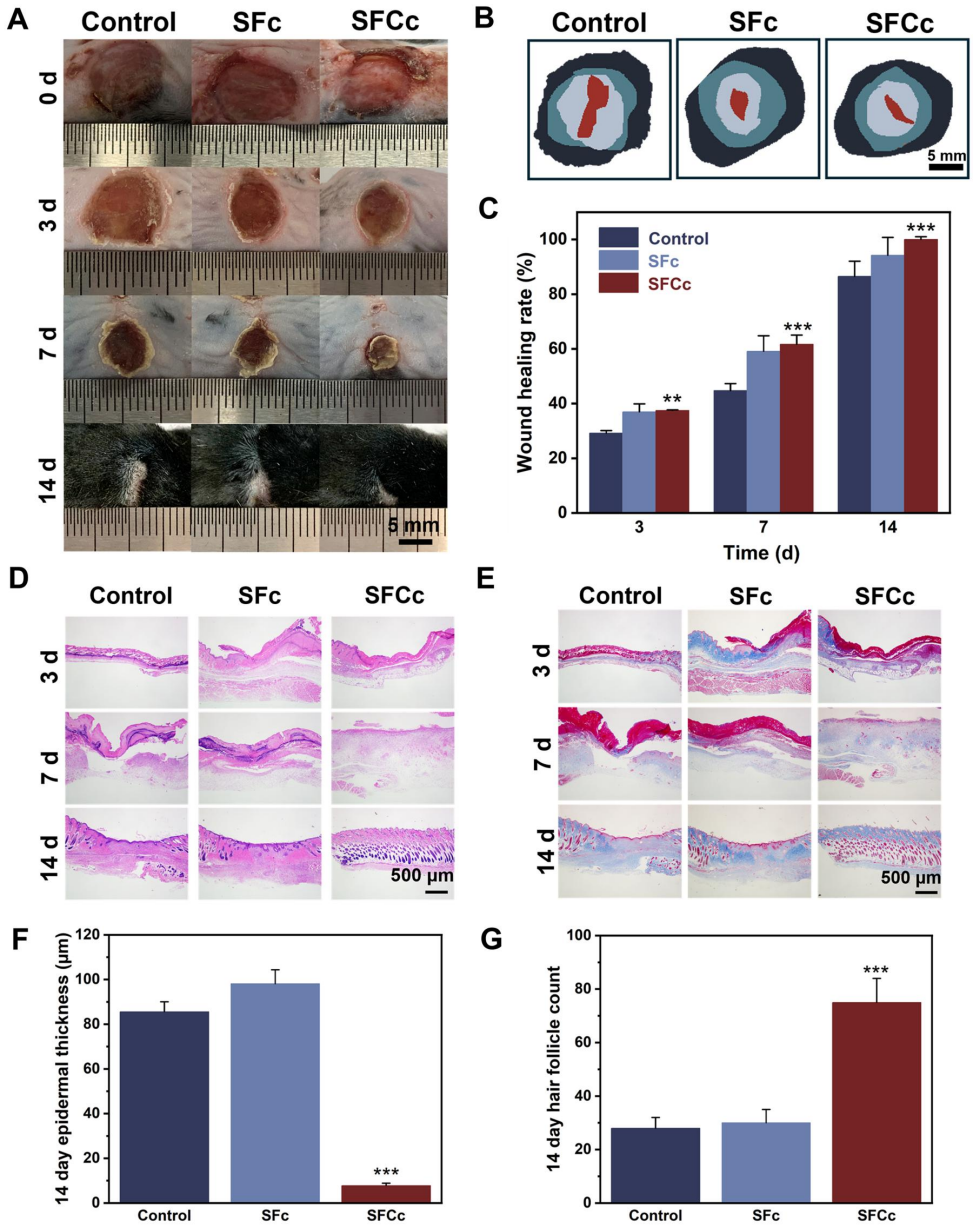
Wound healing assessment and histological analysis. (**A**) Typical images of the wounds over 14 d are illustrated. (**B**) A related integrated diagram shows that the wound region differed at various time points. (**C**) Wound healing rate differed during the 14 d of post-wounding. The data are presented as the mean ± SD. (**D**) H&E staining and (**E**) Masson’s trichrome staining of tissue sections from the wound region on Days 3, 7 and 14. Statistical analyses were conducted on the epidermal thickness (**F**) and number of hair follicles (**G**) over 14 d (**P < 0.01 and ***P < 0.001; *n* = 6).

A mouse pressure ulcer model was established to evaluate the performance of SFCc hydrogels in promoting wound healing (Figure 5A–C). The wound areas of different groups were photographed. The wounds in the control group exhibited suppurative inflammation for 3 d, progressing to only 45% closure by Day 7, with persistent epithelial defects (15% residual wound area) on Day 14. SFc hydrogels displayed intermediate efficacy (91% closure, Day 14), confirming that oxygen-dependent therapeutic enhancement occurred. Wounds treated with SFCc showed no adverse clinical signs (erythema, edema, or suppuration) and achieved complete re-epithelialization by Day 14. At all time points, the wound healing rate was the highest in the SFCc hydrogel group, significantly different from that of the control group. Body weight changes were recorded in mice after surgery. All groups showed an initial decrease followed by an increase in body weight, indicating that wound-related pain affected the appetite of the mice. The changes in the SFC and SFCc groups were like those in the Control group, suggesting that SFC and SFCc did not cause significant systemic toxicity (Supplementary Figure S10).

Histological examinations revealed progressive, phase-specific enhancement of healing metrics in the SFCc-treated wounds (Figure 5D and E). On Days 3 and 7, the wounds in the control group exhibited disorganized extracellular matrix deposition, whereas the wounds in the experimental groups showed significantly enhanced neo-tissue formation. On Day 7, the SFCc group showed smaller wound gaps and more organized collagen fibers with mature cross-linking patterns. By Day 14, all groups showed greater epithelial thickness, but the SFCc group exhibited the greatest increase, with prominent mature glandular lumina and hair follicles (Figure 5F and G). Oxygen-releasing therapy continuously improved the local hypoxic microenvironment of wounds and regulates oxygen-dependent signaling pathways and cellular metabolic states. It promotes the migration and proliferation of keratinocytes, accelerates re-epithelialization and modulates ROS levels and HIF‑1α stability, thereby optimizing angiogenesis and the immune microenvironment. Adequate oxygen supply can activate stem cells in hair follicles and initiate regeneration-related signaling pathways, such as the Wnt/β-catenin pathway, which favor hair follicle neogenesis.

### Enhanced angiogenesis and myofibroblast activity *in vivo* by SFCc hydrogels

**Figure 6:**
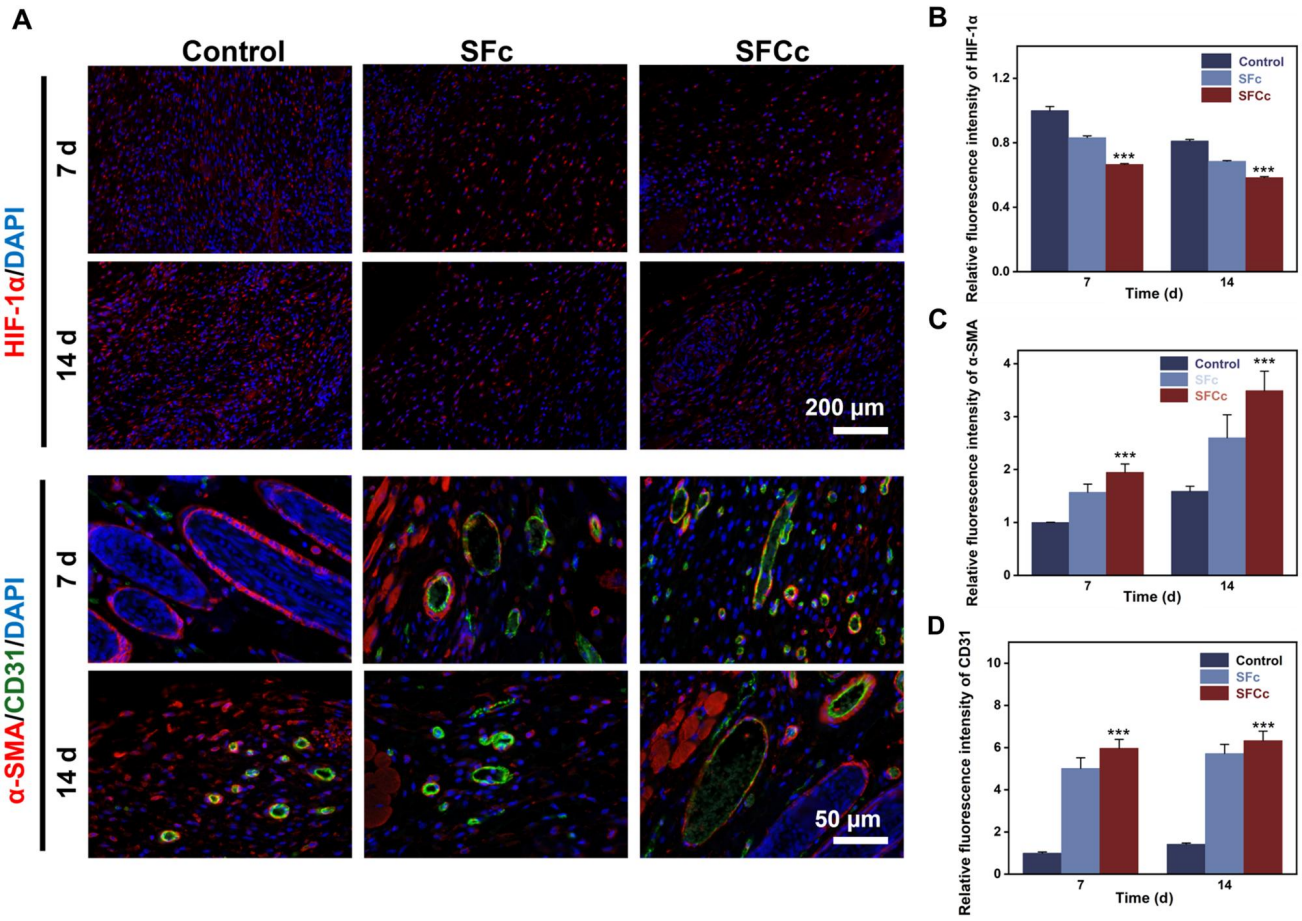
(**A**) Immunofluorescence staining plot and (**B–D**) statistical plot of HIF-1α, α-SMA and CD31 fluorescence intensity (***P < 0.001; *n* = 6).

The recovery of chronic wounds requires angiogenesis, as neovascularization supplies the wound with necessary nutrients and oxygen. Immunofluorescence analysis of the expression of HIF-1α revealed hypoxia resolution patterns across treatment groups (Figure 6A and B). On Days 7 and 14, the wounds in the control group exhibited persistent hypoxia accompanied by intense HIF-1α fluorescence. In contrast, treatment with the SFCc hydrogels resulted in a significant decrease in the expression of the HIF-1α signal after 7 d. The SFc hydrogel showed moderate efficacy, confirming that there was an oxygen dose-dependent effect. This indicated that the SFCc hydrogel is better at resolving hypoxia and promoting vascular adaptation. The superior ability of the SFCc hydrogel to orchestrate wound vascularization and tissue remodeling was demonstrated through coordinated CD31 angiogenesis and activation of α-SMA myofibroblasts (Figure 6C and D). On Day 7, the wounds treated in the SFCc group exhibited a relative CD31 expression of 5.8 ± 0.4, which was 1.2-fold and 5.8-fold higher than the CD31 expression recorded in the SFc group and the control group, respectively. This vascular advantage of SFCc over the SFc and control treatments persisted through 14 d. Concurrently, the expression of α-SMA revealed progressive recruitment of myofibroblasts, with the SFCc hydrogels achieving 1.3-fold and 2.4-fold higher than the control group.

### Modulation of the polarization of macrophages and cytokine expression by SFCc hydrogels in wound healing

**Figure 7:**
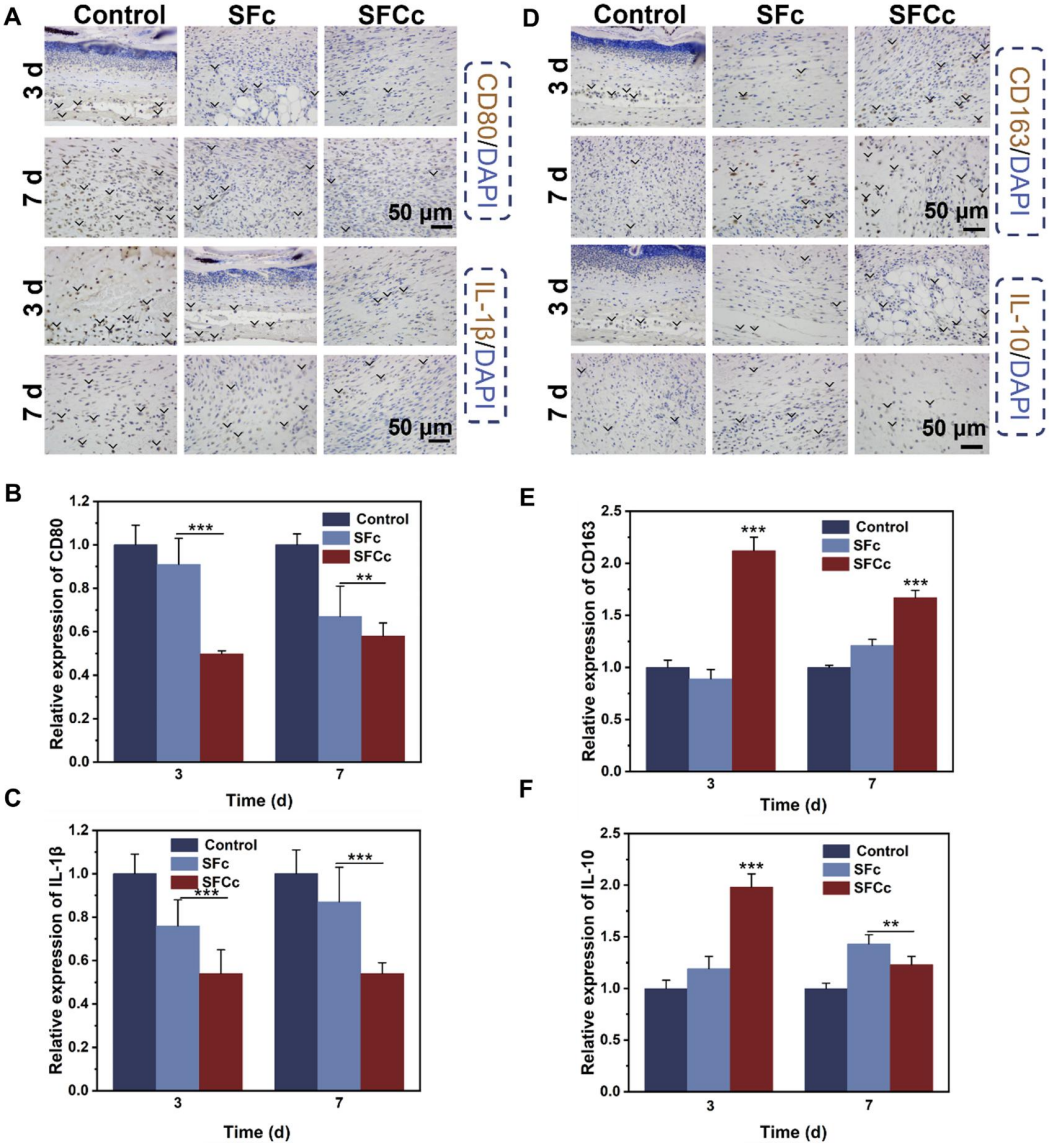
Immunohistochemical staining and quantitative analysis of CD80 and IL-1β (**A–C**), CD163 and IL-10 (**D–F**) (**P < 0.01 and ***P < 0.001; *n* = 6).

Immunohistochemical analysis of macrophage-secreted cytokines revealed that the SFCc hydrogels significantly modulated the inflammatory response during wound healing. The pro-inflammatory factors (CD80 and IL-1β) secreted by M1 macrophages and the anti-inflammatory factors (IL-10 and CD163) secreted by M2 macrophages were assessed via immunohistochemical staining. The expression of IL-1β and CD80 in the wound was lower in the SFCc group than in the control group and the SFc group (Figure 7A–C). On Day 3, the expressions of IL-10 and CD163 in the wounds was higher in the SFCc group than in the control group and the SFc group (Figure 7D–F). These results indicate that SFCc hydrogels effectively modulate macrophage polarization, reducing the levels of pro-inflammatory cytokines and increasing the levels of anti-inflammatory cytokines, thereby increasing the efficiency of wound healing.

We incorporated FeCy, the core electron acceptor, into an implantable hydrogel and evaluated its long-term biosafety *in vivo*. Histological staining of the heart, liver, spleen, lungs and kidneys showed that in both the SFCc and SFc groups, the myocardial fibers were arranged in an orderly manner with no infiltration of inflammatory cells; the hepatocytes appeared normal with no signs of toxic injury. The materials caused no structural damage to the spleen, maintained intact alveolar architecture and preserved the integrity of renal tubular epithelial cells. These results indicated that the concentration of FeCy used in the constructed SFCc and SFc hydrogels exhibits good biocompatibility and does not cause significant acute or subacute toxic effects on the major organs of animals (Supplementary Figure S11) [34, 35].

## Discussion

In this study, we developed a chloroplast-driven platform that synchronizes oxygen supply with metabolic demand through photosynthetic methods, providing a biomimetic solution that performs better than traditional oxygen carriers [36]. The system achieves sustained oxygenation through photosynthetic-chloroplast cores while enabling microenvironment responsive release via pH-sensitive and enzyme-sensitive hydrogels; it represents a significant advancement for applications in chronic wound healing and ischemic tissue repair.

The Hill reaction, a cornerstone of photosynthetic oxygen production, involves the exposure of isolated chloroplasts to light in the presence of an electron acceptor. The efficiency of this reaction is highly sensitive to the choice of Hill oxidant [37, 38]. We found that FeCy outperforms FeCl_3_ in promoting oxygen production, which is consistent with established principles of oxidant specificity [39]. This difference in performance is attributed to the unique pH-modulating properties of FeCy. During photosynthesis, the accumulation of H^+^ ions can decrease the pH, destabilizing chloroplast proteins. However, FeCy undergoes partial decomposition under light to form Fe(OH)_3_, effectively neutralizing excess H^+^ ions and maintaining a stable pH in the environment. This dual functionality of FeCy as an electron acceptor and a pH buffer preserves the structural integrity of chloroplasts and optimizes enzymatic activity, thereby significantly improving oxygen output.

Isolated chloroplasts are vulnerable to ROS, which accumulate under illumination and impair photosynthetic machinery, making it difficult to utilize these chloroplasts. To address this problem, we incorporated Cat to emulate the ROS scavenging capacity found in plant cells. Our results revealed that even minimal Cat supplementation substantially preserved initial DO levels, with higher concentrations achieving peak oxygenation. This dose-dependent increase highlights the dual role of Cat in neutralizing photolytic ROS via the inhibition of the Fenton reaction and sustaining photosynthetic output through the decomposition of H_2_O_2_. The synergistic interplay between the pH-buffered electron transport of FeCy and Cat-mediated scavenging of ROS establishes a robust foundation for long-term photosynthetic oxygen generation, yielding a 3.1-fold increase in cumulative oxygen production compared to conventional systems based on FeCl_3_.

During inflammation, the metabolic demands of immune cells increase dramatically as they migrate from oxygen-rich to hypoxic regions. The HIF pathway plays a pivotal role in modulating the functions of epithelial and immune cells during inflammation, activating adaptive responses in these cells [40]. HIF-1α can promote the polarization of macrophages to the M1 phenotype, enhancing the production of inflammatory chemokines such as TNF-α, IL-1β, IL-6 and IL-8 [41, 42]. Concurrently, HIF-1α acts as a transcriptional regulator of cytokines in macrophages under hypoxic conditions [43]. The SFCc hydrogel reprograms macrophage polarization by providing oxygen to fuel oxidative phosphorylation (OXPHOS) in macrophages, a hallmark of the anti-inflammatory M2 phenotype [44, 45]. Flow cytometry analysis and cytokine profiling revealed significant reductions in the abundance of M1 macrophages and levels of pro-inflammatory cytokines, coupled with a substantial increase in the levels of anti-inflammatory cytokines. This shift toward a regenerative M2-dominated microenvironment is mediated through inhibition of the JAK-STAT3 pathway and activation of SMAD-dependent repair, fostering an environment conducive to wound healing [46].

These coordinated effects suppress hypoxia-induced apoptosis, with SFCc treated cells exhibiting near-physiological apoptosis rates. This outcome is attributed to the hydrogel’s multifunctional capabilities in ROS neutralization, oxygen replenishment and inflammation resolution. By addressing the interrelated challenges of hypoxia, oxidative stress and chronic inflammation, the SFCc hydrogel emerges as a transformative solution for recalcitrant wound healing.

Concurrently, the SFCc hydrogel dynamically reprogrammed macrophage dynamics, transitioning the wound microenvironment from a pro-inflammatory (M1-dominant) state to a regenerative (M2-skewed) state. By 14 d post-treatment, inflammation had resolved, and complete epithelialization was observed, demonstrating the hydrogel’s capacity to transition wounds from a refractory phase to active regeneration. This immunomodulatory effect correlated with suppressed apoptosis rates and enhanced neovascularization, underscoring the hydrogel’s multifunctional therapeutic profile.

This study, however, has certain limitations. While strong light promotes oxygen production, the accompanying heat transfer may increase skin temperature, which could potentially cause tissue damage to some extent. Additionally, although no iron deposition staining was observed in the main organs, the presence of potassium ferricyanide in the body may pose certain risks. Therefore, in future research, we aim to further investigate the balance between light intensity, chlorophyll content and FeCy to achieve high-oxygen production efficiency and biosafety while avoiding damage to normal tissues.

## Conclusions

In this study, we developed SFCc hydrogels for treating chronic wounds. The hydrogel was prepared by integrating the chloroplast Hill reaction system into an *in situ* forming alginate hydrogel. The addition of FeCy significantly increased the oxygen content of the system. Moreover, the incorporation of Cat not only increased oxygen production but also prolonged the activity of isolated chloroplasts. *In vitro* cell experiments showed that high levels of DO, released by chloroplasts in the blood, can alter the hypoxic environment, alleviate cell damage caused by hypoxia, and promote cell proliferation. The SFCc hydrogel also promoted angiogenesis by regulating the expression of CD31 and α-SMA. Additionally, it facilitated the polarization of macrophages toward the M2 phenotype by increasing the levels of anti-inflammatory factors and decreasing the levels of pro-inflammatory factors, thereby accelerating wound healing. Animal experiments confirmed that the SFCc hydrogel effectively promoted wound healing by modulating inflammation and increasing angiogenesis. Overall, we designed a hydrogel dressing capable of *in situ* oxygen delivery at the wound site. This hydrogel can perform multiple functions, including oxygen delivery, angiogenesis, antioxidation and inflammation regulation, offering a novel therapeutic approach for treating chronic wounds. Future studies should focus on further refining the hydrogel formulation and investigating its clinical translation potential to advance the standard of care for patients suffering from chronic wounds.

## Supplementary Material

rbag091_Supplementary_Data

## Data Availability

The data that support the findings of this study are available from the corresponding author on reasonable request.
