# Supplementary material for: Photosynthetic alginate dressing enables sustained oxygen delivery through chloroplast-powered Hill reaction to promote angiogenesis and macrophage reprogramming for chronic wound healing
Source: Regen Biomater. 2026 May 21;13:rbag091. doi: 10.1093/rb/rbag091 (PMC13294445; doi:10.1093/rb/rbag091)
Supplement: rbag091_Supplementary_Data [file rbag091_supplementary_data.zip › SFcc supporting information0417.docx]

**Photosynthetic alginate dressing enables sustained oxygen delivery through chloroplast-powered Hill reaction to promote angiogenesis and macrophage reprogramming for chronic wound healing**

Yifan Zhang^1,2^, Lanqin Yu^2^, Min Fang^2^, Ying Zhou^1^, Kun Zhong^1^, Xinshuo Liu^2^, Xiaoyun Liao^3^, Lihua Li^2*^, Changren Zhou^2^

1. Institute of Dermatology and Venereal Diseases, Affiliated Hospital of Guangdong Medical University, Zhanjiang, 524001, China.

2. College of Chemistry and Materials Science, Engineering Research Center of Artificial Organs and Materials, Jinan University, Guangzhou 510632, China.

3. Guangdong Provincial Key Laboratory of Spine and Spinal Cord Reconstruction, The Fifth Affiliated Hospital (Heyuan Shenhe People’s Hospital), Jinan University, Heyuan 517000, China.

*Corresponding authors:

Professor Lihua Li, Email address: [tlihuali@jnu.edu.cn](mailto:tlihuali@jnu.edu.cn)

Extraction of chloroplasts

Chloroplasts were extracted using differential centrifugation, and fresh spinach was purchased from the market and placed in the refrigerator for 2 days to degrade starch. The spinach leaves were destemmed and cut into pieces of about 1 cm × 1 cm and then loaded into a container. Prepare a chloroplast extract solution containing 400 mmol/L sucrose, 10 mmol/L potassium chloride, 30 mmol/L dibasic sodium phosphate, and 20 mmol/L potassium dibasic phosphate at 0 ℃ and then pour it into the container. The homogenate was homogenized with a high-speed juicer and then was filtered through four layers of cheesecloth. The filtration obtained was centrifuged at 1200 rpm/min for 5 min twice. Subsequently, the supernatant was centrifuged at 4000 rpm/min for an additional 10 min. Then, the extracted chloroplasts are obtained, the precipitate is resuspended in the chloroplast extract solution and stored at 4℃. The extracted chloroplasts were scanned with ultraviolet-visible photometry (Bio-Rad, CFX) in the full wavelength band, and the wavelength band was 300 nm~800 nm.

Morphological analysis

Take 100 μL chloroplast extract and use inverted fluorescence microscope (CKX53, Olympus, Japan) to observe its morphology; 4 μL of chloroplast extract was dropped onto copper mesh, dry and then add 0.5% glutaraldehyde, after 12 h, remove glutaraldehyde, wash twice with sterile water after drying, add ethanol (20%, 40%, 50%, 70%, 90%, 100% per gradient for 6 min) in stages, and the samples were scanned with a transmission electron microscope (Bio-rad, CFX) after natural air drying.

Cytocompatibility analysis of Fecy

CCK-8 assay to assess cell viability

Human Umbilical Vein Endothelial Cells (HUVECs) were seeded into 24-well plates at a density of 1×10⁴ cells per well. After allowing the cells to adhere, culture medium containing different concentrations of ferrocyanide (Fecy; 0.1, 1, and 10 mmol/L) was added to the wells. The cells were then incubated at 37 ℃ for 1, 3, and 5 d. At each point, 30 μL of CCK-8 solution was added to each well, followed by the addition of 270 μL of fresh culture medium. The plates were incubated for an additional 2 h at 37℃ to allow for color development. The absorbance was measured at 450 nm using a spectrophotometric microplate reader (Thermo Fisher Scientific). The viability of the cells was determined by comparing the absorbance values to those of untreated control cells.

Cell Morphology Assessment Using Live-Dead Kit

After 1, 3 and 5 d of incubation with Fecy-containing medium, the cells were washed three times with phosphate-buffered saline (PBS) to remove any residual medium and unbound reagents. Subsequently, 250 μL of Live-Dead staining solution was added to each well, and the cells were incubated for 30 min at room temperature in the dark. Following staining, the cells were observed under a fluorescence microscope (Vert.A1, ZEISS) to assess cell viability and morphology. Live cells were identified by green fluorescence, while dead cells were marked by red fluorescence.

Cytocompatibility analysis of hydrogels

HUVECs cells were seeded into 24-well plates at a density of 1×10^4^ cells per well. The prepared SFCc hydrogel was placed on top of the cells in each well. Subsequently, 600 μL of culture medium was added to each well, and the plates were incubated at 37℃ for 1, 3, and 5 d.

Cell viability and morphology assessment were conducted using CCK-8 assay and Live-dead kit, as detailed in section of Cytocompatibility analysis of Fecy.

Effect of SFCc hydrogel on hypoxic cells

L-929 cells were seeded at a density of 1×10⁴ cells per well in the lower chamber of a 24-well transwell plate and allowed to adhere for 24 h. To induce hypoxia, 200 μM Cobaltous Chloride (CoCl₂) was added to the lower chamber, and the cells were co-incubated for an additional 24 h. Cells cultured in CoCl₂-containing medium served as the hypoxic group, while those cultured in normal medium were designated as the control group. The expression of hypoxia-inducible factors (HIF-1α) in the cells was assessed using Western blot (WB) analysis.

Total proteins were extracted from L-929 cells using the Whole Cell Lysis Assay kit (Beyotime). The extracted proteins were separated on a 10% polyacrylamide gel and subsequently transferred to nitrocellulose membranes using a wet transfer protocol. The membranes were incubated overnight at 4 ℃ with primary antibodies against glyceraldehyde-3-phosphate dehydrogenase (GAPDH) and HIF-1α (Abcam Technology). Following primary antibody incubation, the membranes were treated with anti-rabbit secondary antibodies (Gibco), and the bound antibodies were visualized using Pierce chemiluminescent substrate (Thermo Fisher). The WB results were captured using FLI Capture and analyzed with ImageJ software.

Angiogenesis ability test of hydergels

HUVECs cells were added to the 12-well plate at a density of 3 × 10^5^ cells per well. After the cells reached confluence, a 200 μL pipette tip was used to create uniform scratches on the cell monolayer. The cells were then gently rinsed three times with phosphate-buffered saline (PBS) to remove detached cells. Subsequently, a low serum medium (2% fetal bovine serum, FBS) was added to the wells, and the cells were cultured at 37 ℃. The cells were exposed to light for 15 min every 4 h. Cell migration was monitored at 0, 24, and 48 h using an inverted microscope, and images were captured at each time point. The images were analyzed using ImageJ software to calculate cell mobility and quantify the rate of wound closure. To further evaluate the angiogenic potential, the expression levels of key angiogenesis-related markers, including vascular endothelial growth factor (VEGF), fibroblast growth factor (FGF), and platelet-endothelial cell adhesion molecule (CD31), were measured using real-time fluorescence quantitative PCR (BIO-RAD, CFX). This analysis provided insights into the molecular mechanisms underlying the pro-angiogenic effects of the dressings.

Antioxidant properties of hydrogels

*In vitro* oxidant activity was evaluated by measuring the scavenging rates of four major reactive oxygen species (ROS): 1,1-diphenyl-2-picrylhydrazyl (DPPH), superoxide anion (O^2-^), hydrogen peroxide (H₂O₂), and hydroxyl radical (·OH). The assays were conducted according to the methods previously documented. The test was performed according to the methods previously documented.

Intracellular ROS species scavenging capacity was assessed using 2′,7′-dichlorodihydrofluorescein diacetate (DCFH-DA) probes, which are cell-permeable probes that can be oxidized by ROS to form green, fluorescent dichlorofluorescein (DCF). Cells were stained with DCFH-DA, and the presence of green fluorescence, indicative of ROS, was observed under an inverted fluorescence microscope (CKX53, Olympus, Japan).

Regulatory effects on inflammation

RAW264.7 cells were seeded into 12-well plates at a density of 1×10⁵ cells/mL, with 1 mL of cell suspension added to each well. The cells were incubated for 12 h to allow attachment. Subsequently, the old medium was removed, and 1 μg/mL lipopolysaccharide (LPS) solution was added to transform RAW264.7 cells into the M1 phenotype. After 12 h of LPS treatment, the LPS-containing medium was removed, and the cells were divided into four groups: Control Group (with normal culture medium), Negative Control Group (with medium containing 1 μg/mL LPS), Positive Control Group (with medium supplemented with 20 ng/mL interleukin-4), and Experimental Group (with Hydrogel and normal culture medium).

The plates were then incubated for an additional 48 h. After incubation, cell samples were collected by centrifugation for flow cytometry analysis. The cell supernatants were also collected for enzyme-linked immunosorbent assay (ELISA) analysis (Thermo Fisher Scientific) to measure the levels of cytokines, including IL-6, IL-1β, TGF-β1 and IL-4.

Apoptosis experiments

The apoptosis of cells was assessed using the Annexin V-FITC/propidium iodide (PI) double staining kit. In 6-well plates, 1 mL of cell suspension (at a concentration of 1×10⁵ cells/mL) was added to each well and incubated for 2 h to allow cell attachment. Subsequently, hydrogels were added to the Transwell chamber for 48 h. Cells cultured without hydrogels served as the control group. After 48 h of culture, the cell suspension was collected and centrifuged. The cells were then resuspended in 500 μL of Binding Buffer, followed by the addition of 5 μL of Annexin V-FITC and 5 μL of propidium iodide. The cells were incubated at room temperature in the dark for 10 mi. Finally, the cell suspension was transferred to a flow cytometry tube (Thermo Fisher Scientific) for analysis using a flow cytometer.

**
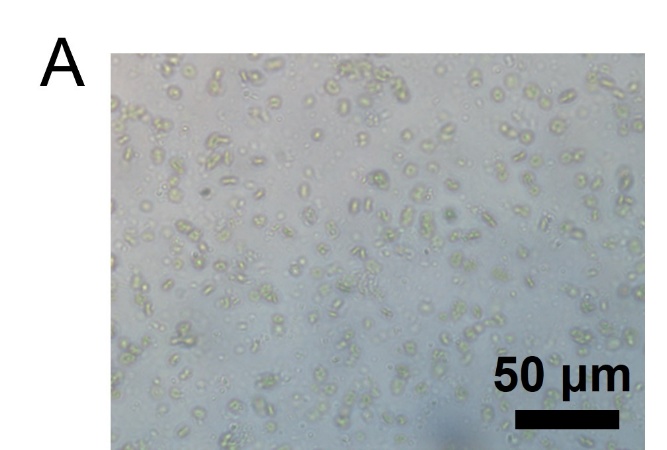
**

**Figure. S1** Chloroplast optical microscope image.


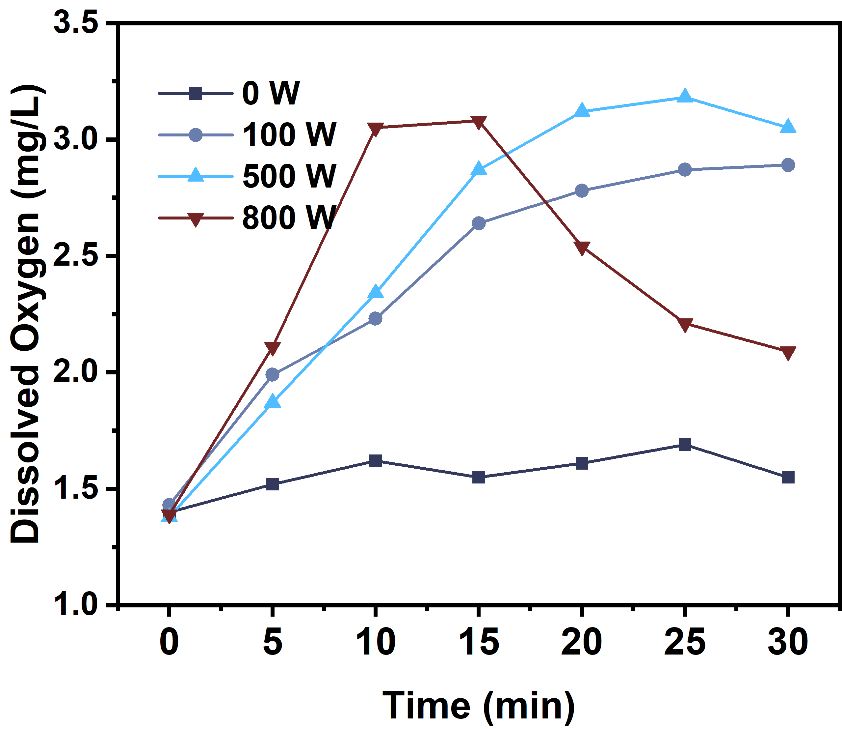


**Figure. S2** Oxygen production test at different power levels

**
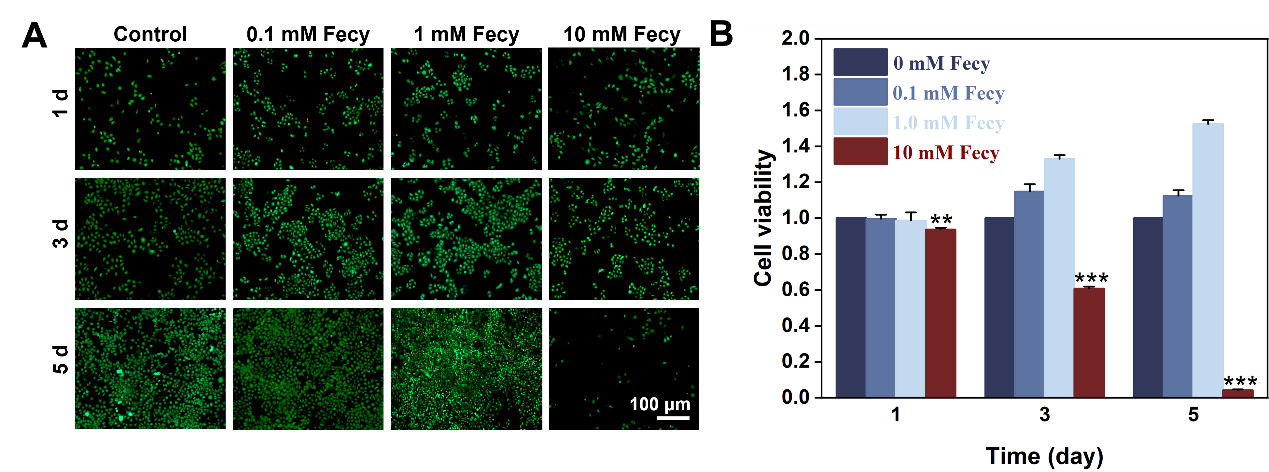
**

**Figure. S3** (A) Cytotoxicity test of different concentrations of Fecy; (B) Cell viability of HUVECs after different concentration Fecy treatment. (**p<0.01, **p<0.001, n=3).


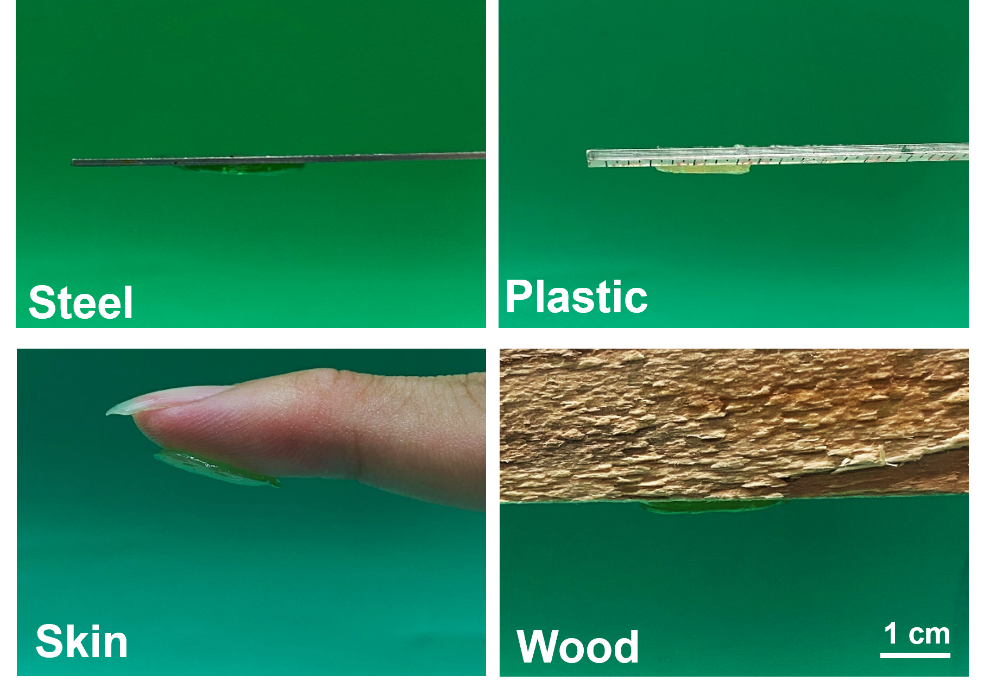


**Figure. S4** Adhesion test of hydrogels.


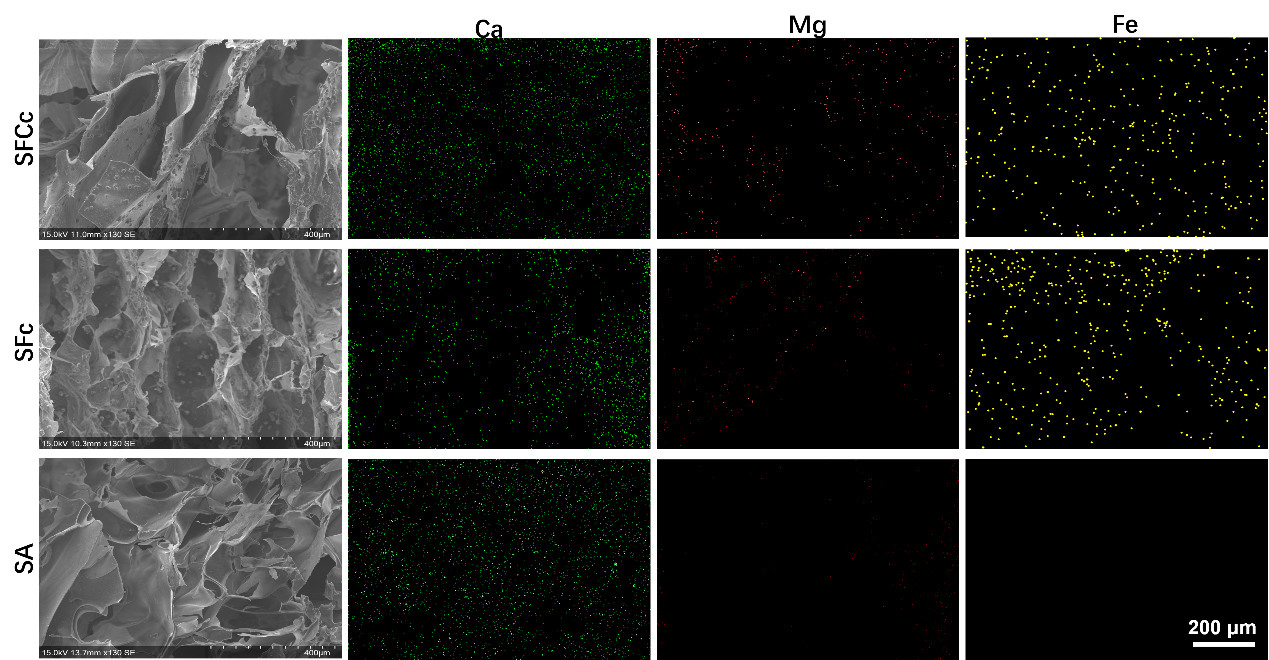


**Figure. S5** SEM and mapping diagrams of SA, SFc and SFCc hydrogels.

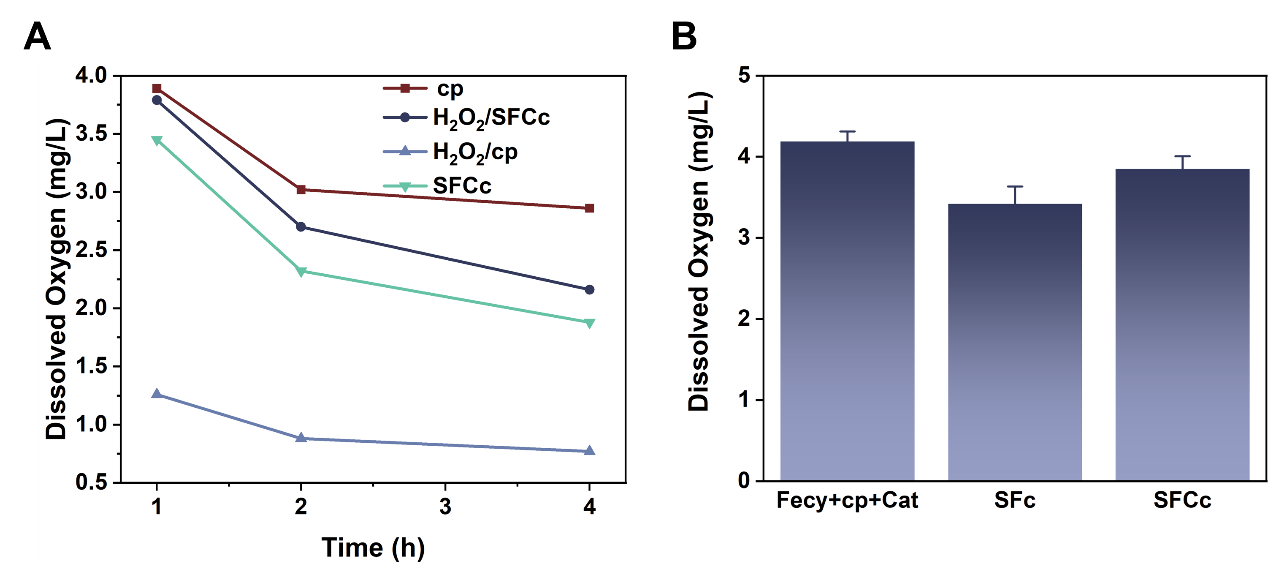


**Figure. S6** (A) Hydrogen peroxide resistance test of hydrogels; (B) Oxygen release capacity test of hydrogels (n=3).


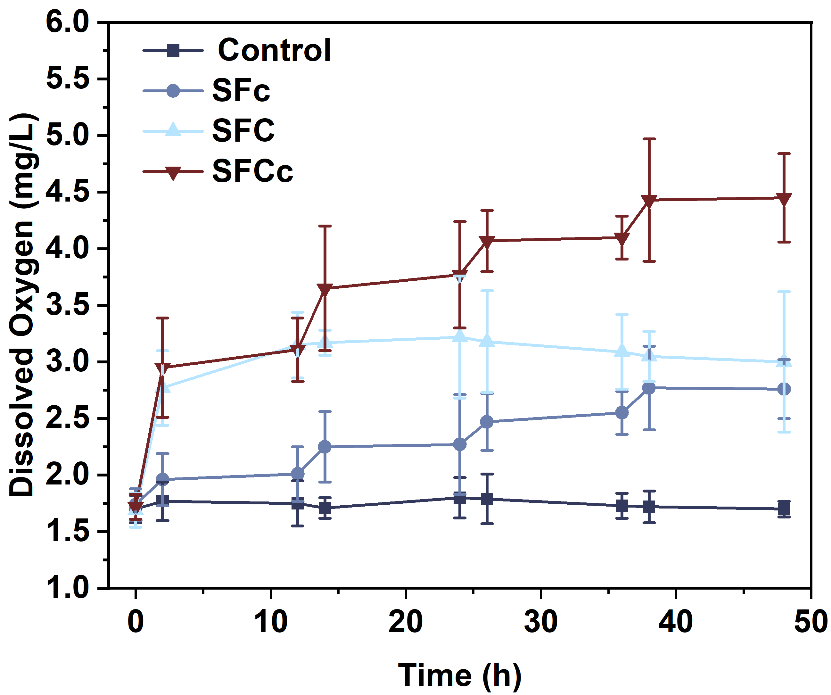


**Figure. S7** Oxygen release kinetics test (n=3).

**
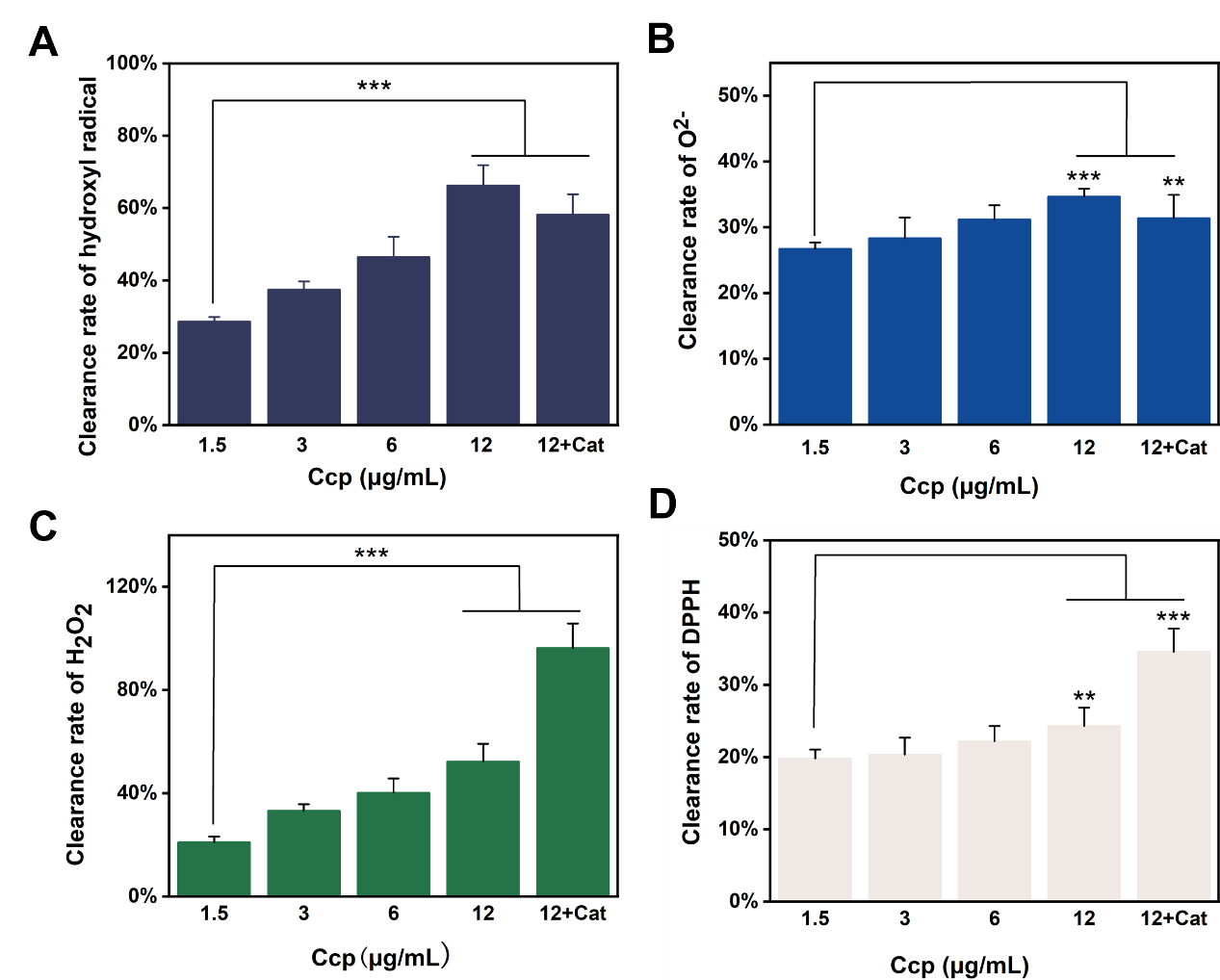
**

**Figure. S8** Scavenging rate of hydrogels’ (A) hydroxyl radical, (B) superoxide anion, (C) hydrogen peroxide scavenging rate and (D) DPPH. (**p<0.01, ***p<0.001, n=3)*.*

**
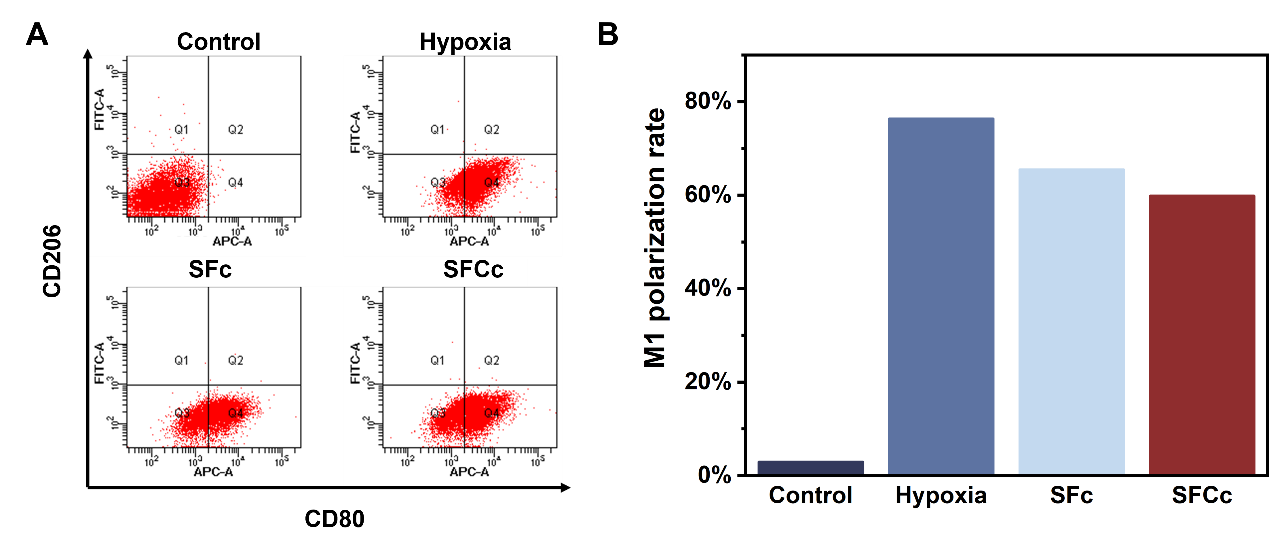
**

**Figure. S9** (A) Flow cytometry results; (B) M1 polarization statistics of RAW264.7 cells after hydrogel treatment.


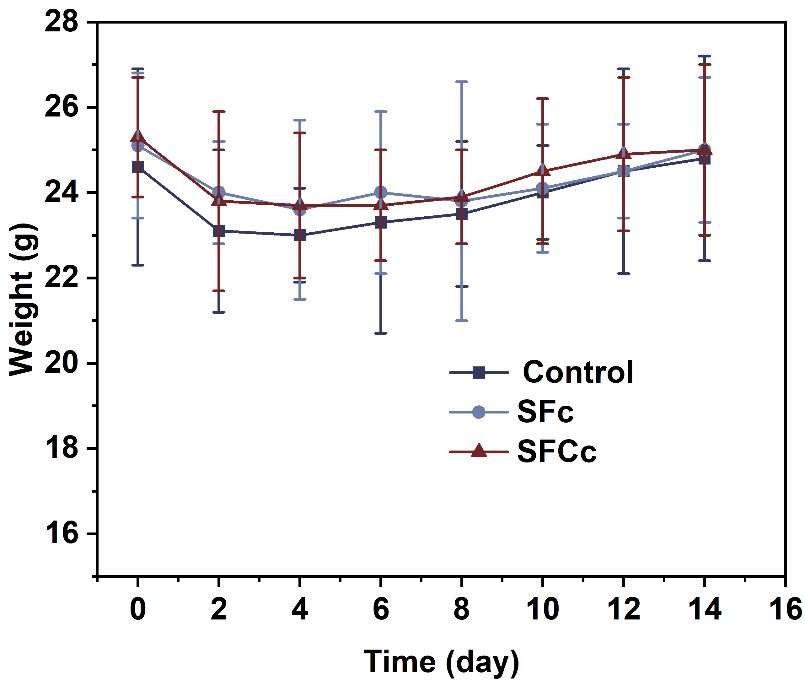


**Figure. S10** Weight changes after mouse surgery.


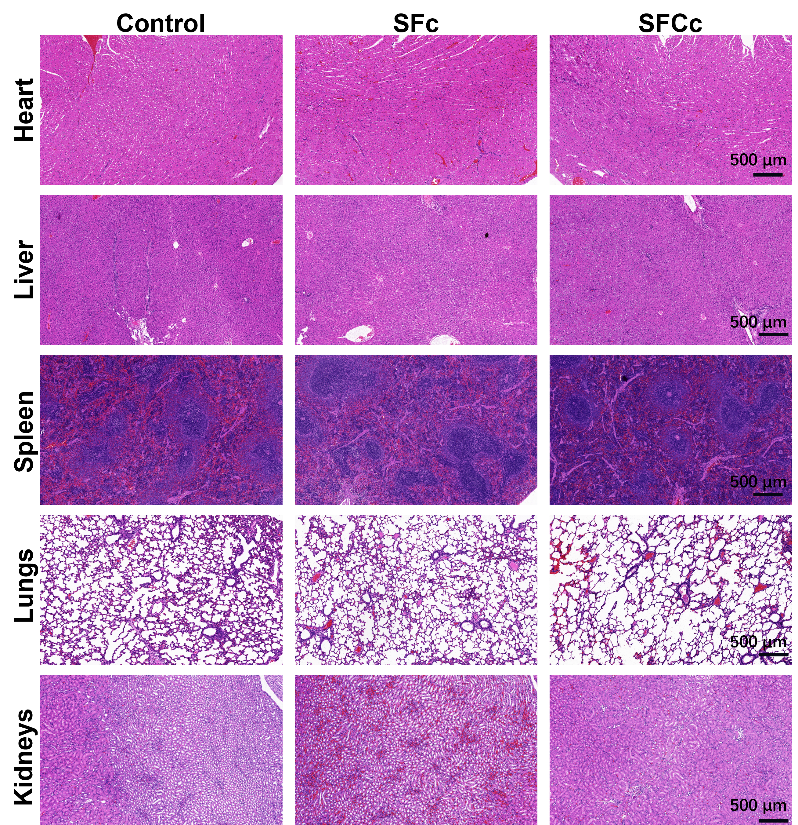


**Figure. S11** H&E analysis of major organs after treatment.
